# Biological function investigated by time-resolved structure determination

**DOI:** 10.1063/4.0000177

**Published:** 2023-02-21

**Authors:** Marius Schmidt

**Affiliations:** Physics Department, University of Wisconsin-Milwaukee, 3135 North Maryland Avenue, Milwaukee, Wisconsin 53211, USA

## Abstract

Inspired by recent progress in time-resolved x-ray crystallography and the adoption of time-resolution by cryo-electronmicroscopy, this article enumerates several approaches developed to become bigger/smaller, faster, and better to gain new insight into the molecular mechanisms of life. This is illustrated by examples where chemical and physical stimuli spawn biological responses on various length and time-scales, from fractions of Ångströms to micro-meters and from femtoseconds to hours.

## RESPONSES OF THE CELLULAR MACHINERY TO STIMULI

All life responds to environmental stimuli. The majority of trees grow leaves in the spring as a response to increasing temperatures and extended sun light. Wounds close after an injury because an army of body cells responds in a concerted fashion. Stimuli, therefore, may have transformative implications on the shape of an organism. Failure in the molecular machinery to perceive and transmit stimuli may impact health and wellbeing in unforeseen ways. Receptors are central to the perception of stimuli. They are located not only in the cell membrane but also in a soluble form in the cytoplasm. They orchestrate finely tuned cellular responses, which are subject to intense investigation not only to cure diseases but also to gain insight in the molecular machinery of cellular organisms.

Present-day grand challenges are outlined by big ideas formulated by the National Science Foundation (NSF) through funding of basic research. They are expressed in a quite general form, which allows for an assortment of diverse ideas and approaches. Here is an outlook on a hypothetical example of the big idea “Understanding the Rules of Life.” When a growth factor binds to its cellular receptor, the entire biochemistry of the cell changes dramatically in preparation of advancing through the cell-cycle (Weinberg, 2007). Its entire machinery can switch from a quiet state to a manufacturing plant that rampantly produces new cellular components and degrades others to get ready for cell division. Since failures in the machinery lead to severe diseases, including cancer (Weinberg, 2007), it is of high interest how this switch is orchestrated, starting from the activation and transcription of distinct DNA sequences to the busy production of new proteins and enzymes. To follow events like these on the molecular level multiple time points, a few seconds apart across a time span of maybe half an hour and across the 3-dimensional volume of an entire single cell are required. Such an approach is very challenging (Patwardhan *et al.*,2014; de Jonge and Peckys, 2016; DiCecco *et al.*, 2021; and Loconte *et al.*, 2023). To observe the molecular rearrangements in real time, the cell must be kept in its physiological environment, and it must survive an imaging process that uses ionizing particles to depict a snapshot of its 3D structure with near atomic resolution. With advances in cryo-electron tomography (Baumeister, 2022), it may be that a 3D view of a cell can be produced also at warm temperatures with sufficient spatial resolution (DiCecco *et al.*, 2021). However, it is not clear whether a living cell can survive an extended period of time while imaged by strongly ionizing particles (electrons) (de Jonge and Peckys, 2016). One may envision that only very limited, low dose data with a very low signal to noise ratio can be collected at each time point, so that the dose deposited is much below a critical level that would otherwise kill the cell. The entire movie of the cell-cycle consists then of multiples of ultra-low dose 3D tomograms that can only be assembled by using mutual information from many if not all snapshots in all orientations and at all time points as demonstrated on other systems using geometric machine learning (Fung *et al.*,2016; Dashti *et al.*, 2020). A successful experiment, however, would reveal the identity, the shape, and the position of a large number of biological macromolecules as a function of time and reveal a deep, hitherto unmatched, insight into the mechanism into cell activation that has numerous bio-medical implications.

## RESPONSES OF INDIVIDUAL BIOLOGICAL MOLECULES TO STIMULI

The function of individual biological macromolecules can be determined from purified preparations with time-resolved structural methods. When crystals can be obtained, time-resolved macromolecular crystallography (TRX) (Moffat, 1989, 2001) is the method of choice since it provides time-resolutions that are commensurate with the time-scales of the reactions to be investigated. Recent advances in single particle cryo-EM allow structures to be determined with near atomic resolution that rivals that of crystallography. However, time-resolved methods need to be implemented that are fast enough to cover macromolecular reactions in a meaningful way (Maeots and Enchev, 2022).

**FIG. 1.**
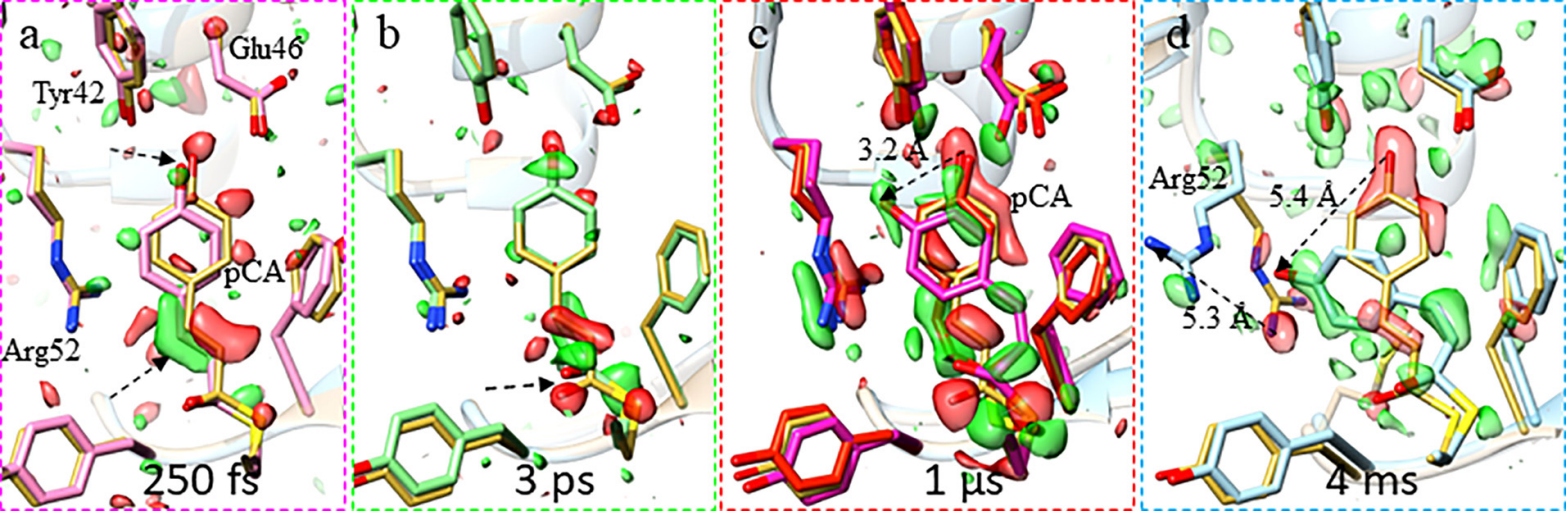
Time-resolved crystallography on photoactive yellow protein. Selected time-delays obtained after initiating the reaction cycle are shown. Difference electron density is depicted by negative (red) and positive (green) features contoured on the 3σ level. The central para-coumaric acid chromophore (pCA) and surrounding amino-acids are marked. The structure of the dark (reference) state is shown in yellow. (a) Femtosecond time-delay. Arrow: displacements are small but pronounced as shown by the strong positive features. The chromophore (pink) is in the *trans*-configuration. (b) Picosecond time-delay: still displacements are quite small (arrow) although the chromophore (green) is in the *cis*-configuration. (c) Microsecond time range. Two intermediates are present as shown by the red and magenta structures. The pCA head is displaced by more than 3 Å (arrow). (d) Millisecond timescale. The pCA is swung out by 5.4 Å (arrow) displacing Arg52 (arrow). Data are from Pande *et al.* (2016) (250 fs and 3 ps), Tenboer *et al.* (2014) (1 *μ*s), and Schmidt *et al.* (2013) (4 ms). The 250 fs, 3 ps, and 1 μs data were collected at the LCLS. The 4 ms data were collected at BioCARS at a temperature of 0 °C as a part of 14 comprehensive time-series spanning a temperature range from −40 to 70 °C.

As TRX at synchrotrons approached maturity (Ren *et al.*,1999; Schotte *et al.*, 2012; Jung *et al.*, 2013; and Schmidt *et al.*, 2013), the advent of x-ray Free Electron Lasers (XFELs) pushed the possible time-resolution to femtoseconds (fs) (Tenboer *et al.*,2014; Barends *et al.*, 2015; Pande *et al.*, 2016; and Nogly *et al.*, 2018), the duration of the x-ray pulses at XFELs. Light stimulated reactions can, therefore, be followed on hitherto unprecedented femtosecond time-scales. In order to ensure uniform illumination, the crystal size should match or be smaller than the absorption length, which is on the order of 5 *μ*m (Tenboer *et al.*,2014; Poddar *et al.*, 2022) for photoactive yellow protein at the absorption maximum. XFEL pulses are capable of producing strong diffraction patterns from micro crystals whose sizes match the absorption length. Ultra short x-ray pulses that enable the “destruction-before-destruction” principle (Chapman *et al.*, 2014) are required to collect a diffraction pattern before the crystal is destroyed. The microcrystal must be replaced with a new, pristine one in a serial fashion. Accordingly, the time-resolved technique has been named ‘time-resolved serial femtosecond crystallography (TR-SFX)’ (Aquila *et al.*, 2012; Tenboer *et al.*, 2014). In order to follow a reaction from the beginning to the end, a time-series of SFX data needs to be collected. Figure 1 shows 4 time points from a time series collected on photoactive yellow protein (PYP) through 11 orders of magnitude from 250 fs to 4 ms. The 4 time points depicted are representative of more than 200 datasets that cover the reaction. These data were collected by multiple groups over a time-period of about 23 years (Genick *et al.*, 1997; Ren *et al.*, 2001; Schmidt *et al.*, 2004; Ihee *et al.*, 2005; Schotte *et al.*, 2012; Jung *et al.*, 2013; Schmidt *et al.*, 2013; Tenboer *et al.*, 2014; Pande *et al.*, 2016; and Pandey *et al.*, 2020) at synchrotrons and XFELs. Around 2010, the speed of TRX data collection at BioCARS (sector 14, Advanced Photon Source, Argonne National Laboratory) has become fast enough that additional parameters such as temperature (Schmidt *et al.*, 2013), pH (Tripathi *et al.*, 2012), or x-ray dose (Schmidt *et al.*, 2012) could be varied in a reasonable amount of experimental time supplementing the time information. This speed, however, will be largely exceeded by high-repetition XFELs, such as the LCLS-II-HE, that are scheduled to continuously generate fs x-ray pulses with a repetition rate of several hundred kHz. With these machines, the collection of several hundred time-delays that densely cover the time range from fs to ms will likely be possible within one experimental shift (12 h).

The amount of digital data generated by the collection of a time-series using an x-ray Free Electron Laser is immense. The stored data of the world had a volume about 300 exabytes (300 × 10^18^ bytes) in 2007 (Hilbert and Lopez, 2011), and it is currently more than 79 000 exabytes (79 zettabytes), which is projected to grow to 160 000 exabytes in 2025 (www.statistica.com). As we are concerned with storage of with our own data, the storage requirement for a time-series is estimated. The time-series might consist of 500 datasets, each dataset at a different time point. For each dataset, about 10 × 10^6^ detector readouts are necessary (most of which do not contain Bragg reflections), which accumulates to 5 × 10^9^ detector readouts for the entire time series. Each detector readout requires 10 Mb of storage. Accordingly, 5 × 10^16^ bytes of storage (50 petabytes) is required. At future high-repetition rate x-ray light sources, on the order of 100 000 detector readouts will be produced per second. The 5 × 10^9^ images required for the time-series are collected in approximately 1 day, which amounts to about 1/40 000 of all data stored worldwide. This is a significant amount. Storage and analysis require immense computational effort that maybe alleviated by discarding diffraction patterns that do not contain Bragg reflections in real time. From an individual point of view, such a scenario is a challenge because a previously enjoyable detector image is nothing more but a glimpse in an ocean of data and not worth too much. Suppose we all can successfully navigate the data ocean, is that what is gained worth such an effort? Let us assume that a sufficiently large representation of a cellular proteome consists of 1000 proteins. A substantial subset of them (maybe 600) can be crystallized, and their structure and function investigated by time-resolved crystallography at a single high-repetition x-ray source in 2 years. This would then provide a database to reconstruct a functionally and structurally meaningful cell content during a cell-cycle as outlined above, a priceless treasure for future drug discovery.

Crystal lattice contacts pose constraints on the dynamics of the crystal's molecules. Accordingly, it would be desirable to cross-check results with another method that provides direct structural information, such as cryo-EM or time-resolved nuclear magnetic resonance (Mock *et al.*, 2017). Imagine that time-resolved cryo-EM would work with sub-millisecond time-resolution consistently with near atomic resolution. This type of data would have an edge over crystallographic data, since rather than originating from an ensemble like a crystal, these data are collected from single particles which would add more dimensions, namely, free energy surfaces (Dashti *et al.*, 2014; Hosseinizadeh *et al.*, 2017), to the results. Yet, time-resolved x-ray crystallography took decades to evolve (Ren *et al.*, 1999; Tenboer *et al.*, 2014) and has the decisive advantage that data can be swiftly collected at (i) physiological temperatures and with (ii) unprecedented time-resolution, both conditions of which are challenge for time-resolved cryo-EM approaches.

In time-resolved crystallography applications, machine learning and artificial intelligence (ML/AI) remain mostly unexplored, although interesting approaches are reported (Schmidt *et al.*, 2003; Ihee *et al.*, 2005; Schmidt *et al.*, 2013; Pande *et al.*, 2016; and Hosseinizadeh *et al.*, 2021). A large challenge remains the deconvolution of kinetic mixtures to extract unique and authentic reaction intermediates in a user-friendly way. In the field of time-resolved spectroscopy, algorithms to extract intermediates are more common (Henry and Hofrichter, 1992; Hoff *et al.*, 1999; Zimanyi *et al.*, 1999a; and Zimanyi *et al.*, 1999b). With the application of the singular value decomposition (an unsupervised machine learning method) to TRX data (Schmidt *et al.*, 2003), one is able to extract the number of processes (intermediates) and their relaxation times by globally identifying them in the right singular vectors. By applying a kinetic model, the structures of the intermediates as well as a compatible kinetic mechanism can also be determined from the left singular vectors (Rajagopal *et al.*, 2004; Schmidt *et al.*, 2004; Ihee *et al.*, 2005; Rajagopal *et al.*, 2005; Jung *et al.*, 2013; and Schmidt *et al.*, 2013). The limitations are that essentially identical concentration profiles of molecules in intermediate states can be produced from an assortment of compatible mechanism. A particular selected mechanism is not unique. However, since similar, chemically sensible concentrations are obtained from multiple degenerate mechanisms, the structures of the intermediates may be, indeed, unique. This must be examined on a case-by-case basis. It is also desirable to streamline the tedious projection algorithm to extract the intermediates from the left singular vectors (Zimanyi *et al.*, 1999b; Schmidt *et al.*, 2003; and Rajagopal *et al.*, 2004). Perhaps, ML/AI methods can be used to extract the intermediates directly from the time-resolved difference maps in a user-friendly way by applying constraints that are a-priori known, such as the number of intermediates (from a prior SVD analysis) and by exploiting the fact that crystallographic occupancy is directly equivalent to (fractional) concentration.

Geometric machine learning methods become interesting when multiple datasets at closely spaced time points can be obtained (Fung *et al.*, 2016; Hosseinizadeh *et al.*, 2021). Spatial completeness is not so much of importance since mutual information from nearby time points can be utilized. In extreme cases, only one (quasi monochromatic) diffraction pattern is necessary per time point (Hosseinizadeh *et al.*, 2021), which covers as little as 0.1% of reciprocal space and which, in addition, is highly partial and, therefore, does not allow for the determination of the required integrated reflection intensities. The information of the remaining ∼20 000 diffraction patterns, which would add up to a crystallographic complete dataset of integrated reflection intensities, can be retrieved from the mutual information extracted from all, however mostly from the closest time points (Hosseinizadeh *et al.*, 2021). It has to be seen how far such an extreme approach carries.

## JABLONSKI DIAGRAMS

**FIG. 2.**
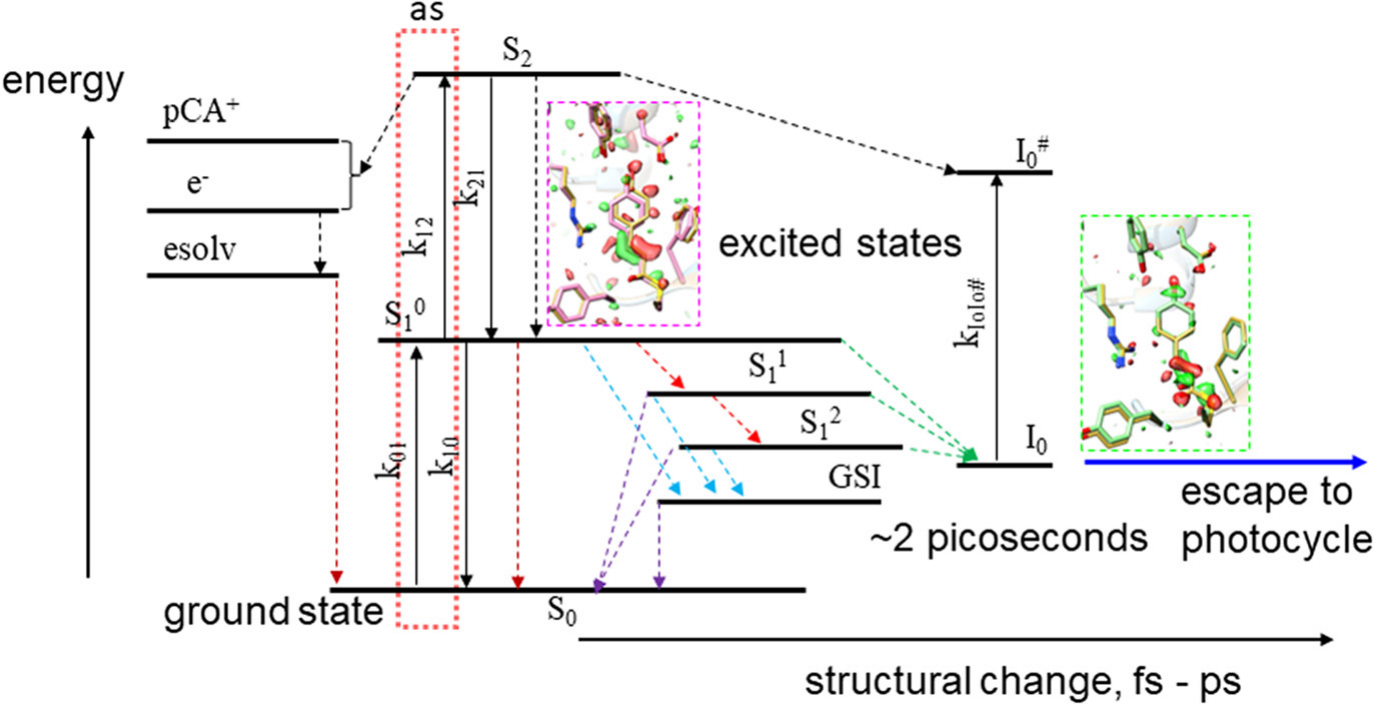
Jablonski diagram of PYP populated with x-ray structures. The Jablonski diagram was developed using ultrafast spectroscopy on PYP (Larsen *et al.*, 2004), extended by Lincoln *et al.* (Lincoln *et al.*, 2012) and interpreted by x-ray structures. Rate coefficients k are Einstein coefficients that determine the probability of absorption and stimulated emission. The dotted box includes ultrafast impulsive absorption events. Dotted arrows are relaxation channels (including spontaneous emission). The S_1_ structure can be associated with the 250 fs structure shown in Fig. 1(a). The structure of I_0_ is similar to the 3 ps structure [see also Fig. 1(b)]. The structure of the ground state intermediate (GSI) is difficult to extract from TR-SFX data. It maybe similar to either I_0_ or to the structure on the S_1_ potential energy surface (PES). Note that structures on S_1_ and S_2_ can relax through several channels to the same photocycle ground state structure I_0_.

The recent ability to determine structures of molecules on a femtosecond timescale after an ultrashort excitation led to the exciting consequence that Jablonski diagrams (Fig. 2) can now be populated by measured (real) x-ray structures (Barends *et al.*, 2015; Nango *et al.*, 2016; Pande *et al.*, 2016; and Nogly *et al.*, 2018). Information obtained from ultrafast absorption, fluorescence, infrared or Raman spectroscopy can be structurally interpreted, and molecular dynamics simulations can be used to further understand structural transitions with simplified models (Groenhof *et al.*, 2002a; Groenhof *et al.*, 2002b; Groenhof, 2013; and Hosseinizadeh *et al.*, 2021). The initial, resonant transitions between the various states in the Jablonski diagram depend on rate coefficients (Einstein coefficients). This way the population change is described by coupled differential equations to accommodate all transition, including absorption and spontaneous and stimulated emission. The initial population dynamics during the reaction initiation is, therefore, dependent on the strength of the light pulse employed, the magnitude, and the frequency dependence of the Einstein coefficients that describe the transition and the time-dependent concentrations of molecules that occupy the Jablonski diagram.

The states in a Jablonski diagram can be considered as time-independent, “metastable” states that are visited by reacting molecules that are vertically excited or de-excited from other time-independent states, mostly from the ground state. With excitation pulses approach a few fs (in the impulsive limit), this picture breaks down. In the established view, an ensemble of molecules is coherently lifted from a ground state potential energy surface to an excited state potential energy surface forming coherent wavepackets (Dhar *et al.*, 1994). The “stable” states predicted by near-equilibrium considerations might not exist on these ultrafast time-scales. Vibrational states can be excited by impulsively displacing the electron cloud relative to the nucleus. The nuclei do not react well to an outside oscillating electromagnetic field (Bruner *et al.*, 2017). However, the electrons will be able to follow the oscillating electric field. Perhaps for a few fs (Bruner *et al.*, 2017), the electrons are not confined to the potential of the nuclei. This means, that for this very short period of time, the so-called Born–Oppenheimer approximation is not valid. In a structural representation, the electrons are substantially displaced by the electromagnetic field of the exciting laser pulse away from the nuclei. If the displacement is not large enough that the electrons can leave the nuclear potential (e.g., as photoelectrons), the electron cloud begins to oscillate shortly thereafter with the positions of the nuclei remaining stationary (Bruner *et al.*, 2017). Accordingly, the excitation process itself becomes complicated in the impulsive limit (Dhar *et al.*, 1994). It might involve transient overshoots, oscillations, and an abrupt electric current that populates high energy unoccupied molecular orbitals with excited (valence) electrons. When the excitation is considered vertical, the structure of the nuclear ensemble does not change. This will produce an energetically unfavorable configuration with the electrons in a new molecular orbital, but the nuclear structure still resembles that of the ground state. However, at this point of time (perhaps a few fs after the excitation event), the Born–Oppenheimer approximation becomes valid again because the electrons are able to quickly adjust to the potential of the nuclei. The atomic configuration relaxes on the excited state energy surface driven by electrostatic forces until they return to the electronic ground state energy surface, in some cases radiation-less through a conical intersection (Groenhof *et al.*, 2004; Levine and Martinez, 2007; Pande *et al.*, 2016; and Hosseinizadeh *et al.*, 2021). Intense, impulsive, ultrashort laser pulses tend to induce damage (Grunbein *et al.*, 2020; Miller *et al.*, 2020). Electrons are accelerated in intense electric fields that may even exceed the electrical breakdown threshold. There is surprisingly little known about the mechanism of absorption, in particular about absorption cross sections as a function of pulse duration (Hutchison *et al.*, 2016). It has been suggested that unspecific damage and the resonance absorption process must be decoupled (Dhar *et al.*, 1994). In addition, there is very little systematic information on how powerful ultrashort laser pulses couple into protein crystals embedded in stabilizing solutions or viscous media. These are all questions that must be addressed in the future.

## ENZYMES

**FIG. 3.**
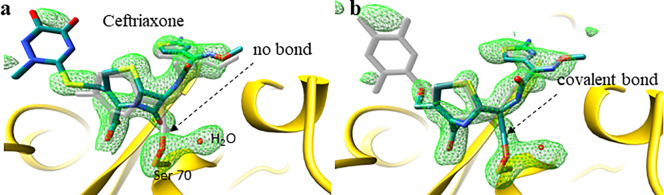
Reaction of β-lactamase with the antibiotics Ceftriaxone (CEF) as revealed by simulated annealing omit maps (green, 2.7 σ contour). Before refinement, the content of the active site (mostly water molecules and a phosphate) was removed, and the Ser70 was treated as a glycine. (a) Formation of a non-covalently bound enzyme-substrate complex at 100 ms MISC delay. Intact CEF is shown in blue, and a putatively covalently bound compound is shown in gray. The arrow shows the gap in the electron density. (b) The covalently bound acyl-intermediate (E–P) is shown in blue, and the intact compound is shown in gray. The electron density at 500 ms MISC delay is continuous across the bond. The β-lactam ring is open. The antibiotic is inactivated.

**FIG. 4.**
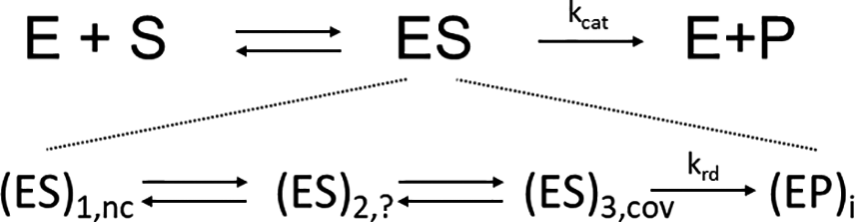
Upper row, steady state, no time-resolution: simplified catalytic mechanism introduced by Michaelis–Menten and Briggs–Haldane (Cornish-Bowden, 2012). The concentration of the enzyme-substrate complex is assumed to be time-independent in the steady state. Second row, transient-state kinetics with time-resolution: substrate binds non-covalently (nc) to the enzyme. The reaction proceeds through intermediates, which may (or may not) consist of covalently (cov) bound substrate. The substrate is catalytically modified to product with several enzyme-product intermediates. A rate determining step parametrized by a rate coefficient k_rd_ determines k_cat_. Arrows depict chemical reactions characterized by rate coefficients.

Enzymes are biological catalysts that catalyze the function of life ranging from digestion of food sources, to motion, perception of stimuli, and cognitive functions. Although their static structures can be determined by x-ray crystallography (Blow and Steitz, 1970) or cryo-EM (Tsai *et al.*, 2022), their functions are difficult to observe directly with atomic precision (Johnson, 2013; Schmidt, 2020; and Wilson, 2022). Methods with time-resolution on enzyme solutions such as absorption spectroscopy in combination with stop-flow mixing experiments (Johnson, 2013) or time-resolved Small and Wide Angle x-ray Scattering (SAXS/WAXS) (Kim *et al.*, 2012; Bjorling *et al.*, 2015; Grant, 2018; and Byer *et al.*, 2022) provide an avenue to investigate enzyme catalysis. However, they all lack atomic resolution. Accordingly, there is uncertainty in the interpretation of the experimental results. One of the most important developments to investigate structures and function of enzymes is the mix-and-inject (Schmidt, 2013) technique that employs diffusion to initiate a reaction (Geremia *et al.*, 2006; Schmidt, 2013). Serial crystallography at intense pulsed x-ray sources in combination with suitable mixing injectors (Calvey *et al.*, 2016; Calvey *et al.*, 2019) enables this technique (Kupitz *et al.*, 2017; Mehrabi *et al.*, 2019a). Since diffusion times dependent on the square of the crystal size, very small crystals must be employed to swiftly initiate enzymatic reactions much faster than the turnover time. Only then can the enzymatic cycle be followed with x-ray structures. Since the crystals are small, already a single exposure results in unacceptable damage by x-ray radiation, so that a new crystal is required. For fastest diffusion, microcrystals with edge lengths on the order of 2 μm are required (Schmidt, 2013). A damage free diffraction pattern might not be obtainable, unless the “diffraction-before-destruction principle” (Chapman *et al.*, 2014) is employed, which requires ultrashort, femtosecond x-ray pulses from XFELs. The method of mixing and then injecting into highly intense pulses from an XFEL has been named mix-and-inject serial crystallography (MISC) (Kupitz *et al.*, 2017). In the last few years, several groundbreaking experiments have been performed to demonstrate the feasibility of MISC (Kupitz *et al.*, 2017; Stagno *et al.*, 2017; Olmos *et al.*, 2018; Dasgupta *et al.*, 2019; Ishigami *et al.*, 2019; Pandey *et al.*, 2021; and Murakawa *et al.*, 2022). More results are expected in the (near) future. With MISC, well-ordered complexes between enzymes and substrates are detected in difference maps (Fig. 3). To reach occupancy values >10% during the initial substrate binding phase, a substrate is required that is soluble to concentrations on the order of that of the crystalline enzyme (∼25 mM). Otherwise, the buildup of the enzyme-substrate complex is too slow and may extend to the steady-state regime even when supported by a high second order binding coefficient. The higher the substrate concentration, the faster the enzyme-substrate complex builds up, and the more time is available to observe intermediates in the burst phase that precedes the steady state. In an extreme case, a substrate concentration of 1 M was used to start reactions in bigger crystals that were investigated with synchrotron pulses (Mehrabi *et al.*, 2019a). With MISC, the initial (pre-steady state) phase of substrate binding and processing is explored. Typically, after formation of a non-covalently bound enzyme-substrate complex, the structures of one or multiple intermediate states may be determined. As one can see from Fig. 4, the steady state Michaelis complex (ES) (Cornish-Bowden, 2012) consists of a mixture of several intermediates weighted by their respective occupancies. Non-covalently and covalently bound enzyme-substrate complexes as well as covalently and non-covalently bound enzyme-product complexes with catalytically modified substrate molecules can all be present at the same time. MISC can be used to unravel the structures of all or a subset of these complexes depending on the kinetic mechanism. As a rule of thumb, only successively more stable intermediates can be observed. Intermediates with a short lifetime cannot be observed after an intermediate with a long lifetime, simply because molecules will not accumulate sufficiently in the short lived intermediate to become observable by MISC (and by any time-resolved method). With MISC, we have a promising and conceptually straightforward method at hand (Schmidt, 2020) that gives insight in enzyme catalysis and ligand binding, which can complement or even replace similar, but technically more challenging approaches using caged substrates (Mehrabi *et al.*, 2019b; Wilamowski *et al.*, 2022).

## SUMMARY

After decades of time-resolved macromolecular structure determination with macroscopically large single crystals, the time for time-resolved serial crystallography that relies on a large number of microscopic crystals has come. Structure and function of an enormous number of biological macromolecules including enzymes can now be determined in real time and at physiological temperatures. New beamlines such as the ID29 SMX instrument at the European Synchrotron Radiation Facility (ESRF) in Grenoble, France, or the PETRA-III 14-2 TREXX instrument at the Deutsches Elektronen Synchrotron (DESY) in Hamburg, Germany, will help to make time-resolved studies with the pump-probe and the mix-and-inject technologies even more popular. Unrivaled time-resolution can be reached at XFELs with very small (even sub-*μ*m) crystals with the benefit of the essential absence of radiation damage. Ultimately, research is carried out by individuals. It is an unwavering interest in time-resolved crystallography that can make a difference.

## Data Availability

Data sharing is not applicable to this article as no new data were created or analyzed in this study.

## References

[1] Aquila, A. , Hunter, M. S. , Doak, R. B. , Kirian, R. A. , Fromme, P. , White, T. A. , Andreasson, J. , Arnlund, D. , Bajt, S. , Barends, T. R. *et al.*, “ Time-resolved protein nanocrystallography using an x-ray free-electron laser,” Opt. Express 20, 2706–2716 (2012). 10.1364/OE.20.002706 22330507 PMC3413412

[2] Barends, T. R. , Foucar, L. , Ardevol, A. , Nass, K. , Aquila, A. , Botha, S. , Doak, R. B. , Falahati, K. , Hartmann, E. , Hilpert, M. *et al.*, “ Direct observation of ultrafast collective motions in CO myoglobin upon ligand dissociation,” Science 350, 445–450 (2015). 10.1126/science.aac5492 26359336

[3] Baumeister, W. , “ Cryo-electron tomography: A long journey to the inner space of cells,” Cell 185, 2649–2652 (2022). 10.1016/j.cell.2022.06.034 35868271

[4] Bjorling, A. , Berntsson, O. , Takala, H. , Gallagher, K. D. , Patel, H. , Gustavsson, E. , St Peter, R. , Duong, P. , Nugent, A. , Zhang, F. *et al.*, “ Ubiquitous structural signaling in bacterial phytochromes,” J. Phys. Chem. Lett. 6, 3379–3383 (2015). 10.1021/acs.jpclett.5b01629 26275765

[5] Blow, D. M. and Steitz, T. A. , “ X-ray diffraction studies of enzymes,” Annu. Rev. Biochem. 39, 63–100 (1970). 10.1146/annurev.bi.39.070170.000431 5479039

[6] Bruner, A. , Hernandez, S. , Mauger, F. , Abanador, P. M. , LaMaster, D. J. , Gaarde, M. B. , Schafer, K. J. , and Lopata, K. , “ Attosecond charge migration with TDDFT: Accurate dynamics from a well-defined initial state,” J. Phys. Chem. Lett. 8, 3991–3996 (2017). 10.1021/acs.jpclett.7b01652 28792225

[7] Byer, A. S. , Pei, X. , Patterson, M. G. , and Ando, N. , “ Small-angle x-ray scattering studies of enzymes,” Curr. Opin. Chem. Biol. 72, 102232 (2022). 10.1016/j.cbpa.2022.102232 36462455 PMC9992928

[8] Calvey, G. D. , Katz, A. M. , and Pollack, L. , “ Microfluidic mixing injector holder enables routine structural enzymology measurements with mix-and-inject serial crystallography using x-ray free electron lasers,” Anal. Chem. 91, 7139–7144 (2019). 10.1021/acs.analchem.9b00311 31060352

[9] Calvey, G. D. , Katz, A. M. , Schaffer, C. B. , and Pollack, L. , “ Mixing injector enables time-resolved crystallography with high hit rate at x-ray free electron lasers,” Struct. Dyn. 3, 054301 (2016). 10.1063/1.4961971 27679802 PMC5010557

[10] Chapman, H. N. , Caleman, C. , and Timneanu, N. , “ Diffraction before destruction,” Philos. Trans. R. Soc., B 369, 20130313 (2014). 10.1098/rstb.2013.0313 PMC4052855 24914146

[11] Cornish-Bowden, A. , *Fundamentals of Enzyme Kinetics*, 4 ed. ( Wiley-VCH, 2012).

[12] Dasgupta, M. , Budday, D. , de Oliveira, S. H. P. , Madzelan, P. , Marchany-Rivera, D. , Seravalli, J. , Hayes, B. , Sierra, R. G. , Boutet, S. , Hunter, M. S. *et al.*, “ Mix-and-inject XFEL crystallography reveals gated conformational dynamics during enzyme catalysis,” Proc. Natl. Acad. Sci. U. S. A. 116, 25634–25640 (2019). 10.1073/pnas.1901864116 31801874 PMC6926069

[13] Dashti, A. , Mashayekhi, G. , Shekhar, M. , Ben Hail, D. , Salah, S. , Schwander, P. , des Georges, A. , Singharoy, A. , Frank, J. , and Ourmazd, A. , “ Retrieving functional pathways of biomolecules from single-particle snapshots,” Nat. Commun. 11, 4734 (2020). 10.1038/s41467-020-18403-x 32948759 PMC7501871

[14] Dashti, A. , Schwander, P. , Langlois, R. , Fung, R. , Li, W. , Hosseinizadeh, A. , Liao, H. Y. , Pallesen, J. , Sharma, G. , Stupina, V. A. *et al.*, “ Trajectories of the ribosome as a Brownian nanomachine,” Proc. Natl. Acad. Sci. U. S. A. 111, 17492–17497 (2014). 10.1073/pnas.1419276111 PMC4267381 25422471

[15] de Jonge, N. and Peckys, D. B. , “ Live cell electron microscopy is probably impossible,” ACS Nano 10, 9061–9063 (2016). 10.1021/acsnano.6b02809 27776410

[16] Dhar, L. , Rogers, J. A. , and Nelson, K. A. , “ Time-resolved vibrational spectroscopy in the impulsive limit,” Chem. Rev. 94, 157–193 (1994). 10.1021/cr00025a006

[17] DiCecco, L. A. , D'Elia, A. , Miller, C. , Sask, K. N. , Soleymani, L. , and Grandfield, K. , “ Electron microscopy imaging applications of room temperature ionic liquids in the biological field: A review,” Chembiochem 22, 2488–2506 (2021). 10.1002/cbic.202100041 33690961

[18] Fung, R. , Hanna, A. M. , Vendrell, O. , Ramakrishna, S. , Seideman, T. , Santra, R. , Ourmazd, A. , and Ourmazd, A. , “ Dynamics from noisy data with extreme timing uncertainty,” Nature 532, 471–475 (2016). 10.1038/nature17627 27121840

[19] Genick, U. K. , Borgstahl, G. E. , Ng, K. , Ren, Z. , Pradervand, C. , Burke, P. M. , Srajer, V. , Teng, T. Y. , Schildkamp, W. , McRee, D. E. *et al.*, “ Structure of a protein photocycle intermediate by millisecond time-resolved crystallography,” Science 275, 1471–1475 (1997). 10.1126/science.275.5305.1471 9045611

[20] Geremia, S. , Campagnolo, M. , Demitri, N. , and Johnson, L. N. , “ Simulation of diffusion time of small molecules in protein crystals,” Structure 14, 393–400 (2006). 10.1016/j.str.2005.12.007 16531224

[21] Grant, T. D. , “ Ab initio electron density determination directly from solution scattering data,” Nat. Methods 15, 191–193 (2018). 10.1038/nmeth.4581 29377013

[22] Groenhof, G. , “ Introduction to QM/MM Simulations,” Methods Mol. Biol. 924, 43–66 (2013). 10.1007/978-1-62703-017-5 23034745

[23] Groenhof, G. , Bouxin-Cademartory, M. , Hess, B. , De Visser, S. P. , Berendsen, H. J. , Olivucci, M. , Mark, A. E. , and Robb, M. A. , “ Photoactivation of the photoactive yellow protein: Why photon absorption triggers a trans-to-cis Isomerization of the chromophore in the protein,” J. Am. Chem. Soc. 126, 4228–4233 (2004). 10.1021/ja039557f 15053611

[24] Groenhof, G. , Lensink, M. F. , Berendsen, H. J. , and Mark, A. E. , “ Signal transduction in the photoactive yellow protein. II. Proton transfer initiates conformational changes,” Proteins 48, 212–219 (2002a). 10.1002/prot.10135 12112690

[25] Groenhof, G. , Lensink, M. F. , Berendsen, H. J. , Snijders, J. G. , and Mark, A. E. , “ Signal transduction in the photoactive yellow protein. I. Photon absorption and the isomerization of the chromophore,” Proteins 48, 202–211 (2002b). 10.1002/prot.10136 12112689

[26] Grunbein, M. L. , Stricker, M. , Nass Kovacs, G. , Kloos, M. , Doak, R. B. , Shoeman, R. L. , Reinstein, J. , Lecler, S. , Haacke, S. , and Schlichting, I. , “ Illumination guidelines for ultrafast pump-probe experiments by serial femtosecond crystallography,” Nat. Methods 17, 681–684 (2020). 10.1038/s41592-020-0847-3 32451477

[27] Henry, E. R. and Hofrichter, J. , “ Singular value decomposition: Application to analysis of experimental-data,” Methods Enzymol. 210, 129–192 (1992). 10.1016/0076-6879(92)10010-B

[28] Hilbert, M. and Lopez, P. , “ The world's technological capacity to store, communicate, and compute information,” Science 332, 60–65 (2011). 10.1126/science.1200970 21310967

[29] Hoff, W. D. , Xie, A. , Van Stokkum, I. H. , Tang, X. J. , Gural, J. , Kroon, A. R. , and Hellingwerf, K. J. , “ Global conformational changes upon receptor stimulation in photoactive yellow protein,” Biochemistry 38, 1009–1017 (1999). 10.1021/bi980504y 9893997

[30] Hosseinizadeh, A. , Breckwoldt, N. , Fung, R. , Sepehr, R. , Schmidt, M. , Schwander, P. , Santra, R. , and Ourmazd, A. , “ Few-fs resolution of a photoactive protein traversing a conical intersection,” Nature 599, 697 (2021). 10.1038/s41586-021-04050-9 34732893

[31] Hosseinizadeh, A. , Mashayekhi, G. , Copperman, J. , Schwander, P. , Dashti, A. , Sepehr, R. , Fung, R. , Schmidt, M. , Yoon, C. H. , Hogue, B. G. *et al.*, “ Conformational landscape of a virus by single-particle x-ray scattering,” Nat. Methods 14, 877–881 (2017). 10.1038/nmeth.4395 28805793

[32] Hutchison, C. D. M. , Kaucikas, M. , Tenboer, J. , Kupitz, C. , Moffat, K. , Schmidt, M. , and van Thor, J. J. , “ Photocycle populations with femtosecond excitation of crystalline photoactive yellow protein,” Chem. Phys. Lett. 654, 63–71 (2016). 10.1016/j.cplett.2016.04.087

[33] Ihee, H. , Rajagopal, S. , Srajer, V. , Pahl, R. , Anderson, S. , Schmidt, M. , Schotte, F. , Anfinrud, P. A. , Wulff, M. , and Moffat, K. , “ Visualizing reaction pathways in photoactive yellow protein from nanoseconds to seconds,” Proc. Natl. Acad. Sci. U. S. A. 102, 7145–7150 (2005). 10.1073/pnas.0409035102 15870207 PMC1088170

[34] Ishigami, I. , Lewis-Ballester, A. , Echelmeier, A. , Brehm, G. , Zatsepin, N. A. , Grant, T. D. , Coe, J. D. , Lisova, S. , Nelson, G. , Zhang, S. *et al.*, “ Snapshot of an oxygen intermediate in the catalytic reaction of cytochrome c oxidase,” Proc. Natl. Acad. Sci. U. S. A. 116, 3572–3577 (2019). 10.1073/pnas.1814526116 PMC6397517 30808749

[35] Johnson, K. A. , “ A century of enzyme kinetic analysis, 1913 to 2013,” FEBS Lett. 587, 2753–2766 (2013). 10.1016/j.febslet.2013.07.012 23850893 PMC4624389

[36] Jung, Y. O. , Lee, J. H. , Kim, J. , Schmidt, M. , Moffat, K. , Srajer, V. , and Ihee, H. , “ Volume-conserving trans-cis isomerization pathways in photoactive yellow protein visualized by picosecond x-ray crystallography,” Nat. Chem. 5, 212–220 (2013). 10.1038/nchem.1565 23422563 PMC3579544

[37] Kim, T. W. , Lee, J. H. , Choi, J. , Kim, K. H. , van Wilderen, L. J. , Guerin, L. , Kim, Y. , Jung, Y. O. , Yang, C. , Kim, J. *et al.*, “ Protein structural dynamics of photoactive yellow protein in solution revealed by pump-probe x-ray solution scattering,” J. Am. Chem. Soc. 134, 3145–3153 (2012). 10.1021/ja210435n 22304441

[38] Kupitz, C. , Olmos, Jr., J. L. , Holl, M. , Tremblay, L. , Pande, K. , Pandey, S. , Oberthur, D. , Hunter, M. , Liang, M. , Aquila, A. *et al.*, “ Structural enzymology using x-ray free electron lasers,” Struct. Dyn. 4, 044003 (2017). 10.1063/1.4972069 28083542 PMC5178802

[39] Larsen, D. S. , van Stokkum, I. H. , Vengris, M. , van Der Horst, M. A. , de Weerd, F. L. , Hellingwerf, K. J. , and van Grondelle, R. , “ Incoherent manipulation of the photoactive yellow protein photocycle with dispersed pump-dump-probe spectroscopy,” Biophys. J. 87, 1858–1872 (2004). 10.1529/biophysj.104.043794 15345564 PMC1304590

[40] Levine, B. G. and Martinez, T. J. , “ Isomerization through conical intersections,” Annu. Rev. Phys. Chem. 58, 613–634 (2007). 10.1146/annurev.physchem.57.032905.104612 17291184

[41] Lincoln, C. N. , Fitzpatrick, A. E. , and van Thor, J. J. , “ Photoisomerisation quantum yield and non-linear cross-sections with femtosecond excitation of the photoactive yellow protein,” Phys. Chem. Chem. Phys. 14, 15752–15764 (2012). 10.1039/c2cp41718a 23090503

[42] Loconte, V. , Chen, J. H. , Vanslembrouck, B. , Ekman, A. A. , McDermott, G. , Le Gros, M. A. , and Larabell, C. A. , “ Soft x-ray tomograms provide a structural basis for whole-cell modeling,” FASEB J. 37, e22681 (2023). 10.1096/fj.202200253R 36519968 PMC10107707

[43] Maeots, M. E. and Enchev, R. I. , “ Structural dynamics: Review of time-resolved cryo-EM,” Acta Crystallogr., Sect. D 78, 927–935 (2022). 10.1107/S2059798322006155 PMC9344476 35916218

[44] Mehrabi, P. , Schulz, E. C. , Agthe, M. , Horrell, S. , Bourenkov, G. , von Stetten, D. , Leimkohl, J. P. , Schikora, H. , Schneider, T. R. , Pearson, A. R. *et al.*, “ Liquid application method for time-resolved analyses by serial synchrotron crystallography,” Nat. Methods 16, 979 (2019a). 10.1038/s41592-019-0553-1 31527838

[45] Mehrabi, P. , Schulz, E. C. , Dsouza, R. , Muller-Werkmeister, H. M. , Tellkamp, F. , Miller, R. J. D. , and Pai, E. F. , “ Time-resolved crystallography reveals allosteric communication aligned with molecular breathing,” Science 365, 1167–1170 (2019b). 10.1126/science.aaw9904 31515393

[46] Miller, R. J. D. , Pare-Labrosse, O. , Sarracini, A. , and Besaw, J. E. , “ Three-dimensional view of ultrafast dynamics in photoexcited bacteriorhodopsin in the multiphoton regime and biological relevance,” Nat. Commun. 11, 1240 (2020). 10.1038/s41467-020-14971-0 32144255 PMC7060340

[47] Mock, J. Y. , Xu, Y. , Ye, Y. , and Clemons, Jr., W. M. , “ Structural basis for regulation of the nucleo-cytoplasmic distribution of Bag6 by TRC35,” Proc. Natl. Acad. Sci. U. S. A. 114, 11679–11684 (2017). 10.1073/pnas.1702940114 PMC5676875 29042515

[48] Moffat, K. , “ Time-resolved macromolecular crystallography,” Annu. Rev. Biophys. Biophys. Chem. 18, 309–332 (1989). 10.1146/annurev.bb.18.060189.001521 2660828

[49] Moffat, K. , “ Time-resolved biochemical crystallography: A mechanistic perspective,” Chem. Rev. 101, 1569–1581 (2001). 10.1021/cr990039q 11709992

[50] Murakawa, T. , Suzuki, M. , Fukui, K. , Masuda, T. , Sugahara, M. , Tono, K. , Tanaka, T. , Iwata, S. , Nango, E. , Yano, T. *et al.*, “ Serial femtosecond x-ray crystallography of an anaerobically formed catalytic intermediate of copper amine oxidase,” Acta Crystallogr., Sect. D 78, 1428–1438 (2022). 10.1107/S2059798322010385 36458614

[51] Nango, E. , Royant, A. , Kubo, M. , Nakane, T. , Wickstrand, C. , Kimura, T. , Tanaka, T. , Tono, K. , Song, C. Y. , Tanaka, R. *et al.*, “ A three-dimensional movie of structural changes in bacteriorhodopsin,” Science 354, 1552–1557 (2016). 10.1126/science.aah3497 28008064

[52] Nogly, P. , Weinert, T. , James, D. , Carbajo, S. , Ozerov, D. , Furrer, A. , Gashi, D. , Borin, V. , Skopintsev, P. , Jaeger, K. *et al.*, “ Retinal isomerization in bacteriorhodopsin captured by a femtosecond x-ray laser,” Science 361, eaat0094 (2018). 10.1126/science.aat0094 29903883

[53] Olmos, Jr., J. L. , Pandey, S. , Martin-Garcia, J. M. , Calvey, G. , Katz, A. , Knoska, J. , Kupitz, C. , Hunter, M. S. , Liang, M. , Oberthuer, D. *et al.*, “ Enzyme intermediates captured ‘on the fly’ by mix-and-inject serial crystallography,” BMC Biol. 16, 59 (2018). 10.1186/s12915-018-0524-5 29848358 PMC5977757

[54] Pande, K. , Hutchison, C. D. M. , Groenhof, G. , Aquila, A. , Robinson, J. S. , Tenboer, J. , Basu, S. , Boutet, S. , Deponte, D. , Liang, M. *et al.*, “ Femtosecond structural dynamics drives the trans/cis isomerization in photoactive yellow protein,” Science 352, 725–729 (2016). 10.1126/science.aad5081 27151871 PMC5291079

[55] Pandey, S. , Bean, R. , Sato, T. , Poudyal, I. , Bielecki, J. , Cruz Villarreal, J. , Yefanov, O. , Mariani, V. , White, T. A. , Kupitz, C. *et al.*, “ Time-resolved serial femtosecond crystallography at the European XFEL,” Nat. Methods 17, 73–78 (2020). 10.1038/s41592-019-0628-z 31740816 PMC9113060

[56] Pandey, S. , Calvey, G. , Katz, A. M. , Malla, T. N. , Koua, F. H. M. , Martin-Garcia, J. M. , Poudyal, I. , Yang, J. H. , Vakili, M. , Yefanov, O. *et al.*, “ Observation of substrate diffusion and ligand binding in enzyme crystals using high-repetition-rate mix-and-inject serial crystallography,” IUCrJ 8, 878–895 (2021). 10.1107/S2052252521008125 PMC8562667 34804542

[57] Patwardhan, A. , Ashton, A. , Brandt, R. , Butcher, S. , Carzaniga, R. , Chiu, W. , Collinson, L. , Doux, P. , Duke, E. , Ellisman, M. H. *et al.*, “ A 3D cellular context for the macromolecular world,” Nat. Struct. Mol. Biol. 21, 841–845 (2014). 10.1038/nsmb.2897 25289590 PMC4346196

[58] Poddar, H. , Heyes, D. J. , Schiro, G. , Weik, M. , Leys, D. , and Scrutton, N. S. , “ A guide to time-resolved structural analysis of light-activated proteins,” FEBS J. 289, 576–595 (2022). 10.1111/febs.15880 33864718

[59] Rajagopal, S. , Anderson, S. , Srajer, V. , Schmidt, M. , Pahl, R. , and Moffat, K. , “ A structural pathway for signaling in the E46Q mutant of photoactive yellow protein,” Structure 13, 55–63 (2005). 10.1016/j.str.2004.10.016 15642261

[60] Rajagopal, S. , Schmidt, M. , Anderson, S. , Ihee, H. , and Moffat, K. , “ Analysis of experimental time-resolved crystallographic data by singular value decomposition,” Acta Crystallogr., Sect. D 60, 860–871 (2004). 10.1107/S0907444904004160 15103131

[61] Ren, Z. , Bourgeois, D. , Helliwell, J. R. , Moffat, K. , Srajer, V. , and Stoddard, B. L. , “ Laue crystallography: Coming of age,” J. Synchrotron Radiat. 6, 891–917 (1999). 10.1107/S0909049599006366

[62] Ren, Z. , Perman, B. , Srajer, V. , Teng, T. Y. , Pradervand, C. , Bourgeois, D. , Schotte, F. , Ursby, T. , Kort, R. , Wulff, M. *et al.*, “ A molecular movie at 1.8 A resolution displays the photocycle of photoactive yellow protein, a eubacterial blue-light receptor, from nanoseconds to seconds,” Biochemistry 40, 13788–13801 (2001). 10.1021/bi0107142 11705368

[63] Schmidt, M. , “ Mix and inject, reaction initiation by diffusion for time-resolved macromolecular crystallography,” Adv. Condens. Matter Phys. 2013, 167276. 10.1155/2013/167276

[64] Schmidt, M. , “ Reaction initiation in enzyme crystals by diffusion of substrate,” Crystals 10, 116 (2020). 10.3390/cryst10020116

[65] Schmidt, M. , Pahl, R. , Srajer, V. , Anderson, S. , Ren, Z. , Ihee, H. , Rajagopal, S. , and Moffat, K. , “ Protein kinetics: Structures of intermediates and reaction mechanism from time-resolved x-ray data,” Proc. Natl. Acad. Sci. U. S. A. 101, 4799–4804 (2004). 10.1073/pnas.0305983101 15041745 PMC387328

[66] Schmidt, M. , Rajagopal, S. , Ren, Z. , and Moffat, K. , “ Application of singular value decomposition to the analysis of time-resolved macromolecular x-ray data,” Biophys. J. 84, 2112–2129 (2003). 10.1016/S0006-3495(03)75018-8 12609912 PMC1302779

[67] Schmidt, M. , Srajer, V. , Henning, R. , Ihee, H. , Purwar, N. , Tenboer, J. , and Tripathi, S. , “ Protein energy landscapes determined by five-dimensional crystallography,” Acta Crystallogr., Sect. D 69, 2534–2542 (2013). 10.1107/S0907444913025997 24311594 PMC3852658

[68] Schmidt, M. , Srajer, V. , Purwar, N. , and Tripathi, S. , “ The kinetic dose limit in room-temperature time-resolved macromolecular crystallography,” J. Synchrotron Radiat. 19, 264–273 (2012). 10.1107/S090904951105549X 22338689 PMC3284346

[69] Schotte, F. , Cho, H. S. , Kaila, V. R. , Kamikubo, H. , Dashdorj, N. , Henry, E. R. , Graber, T. J. , Henning, R. , Wulff, M. , Hummer, G. *et al.*, “ Watching a signaling protein function in real time via 100-ps time-resolved Laue crystallography,” Proc. Natl. Acad. Sci. U. S. A. 109, 19256–19261 (2012). 10.1073/pnas.1210938109 PMC3511082 23132943

[70] Stagno, J. R. , Liu, Y. , Bhandari, Y. R. , Conrad, C. E. , Panja, S. , Swain, M. , Fan, L. , Nelson, G. , Li, C. , Wendel, D. R. *et al.*, “ Structures of riboswitch RNA reaction states by mix-and-inject XFEL serial crystallography,” Nature 541, 242–246 (2017). 10.1038/nature20599 27841871 PMC5502819

[71] Tenboer, J. , Basu, S. , Zatsepin, N. , Pande, K. , Milathianaki, D. , Frank, M. , Hunter, M. , Boutet, S. , Williams, G. J. , Koglin, J. E. *et al.*, “ Time-resolved serial crystallography captures high-resolution intermediates of photoactive yellow protein,” Science 346, 1242–1246 (2014). 10.1126/science.1259357 25477465 PMC4361027

[72] Tripathi, S. , Srajer, V. , Purwar, N. , Henning, R. , and Schmidt, M. , “ pH dependence of the photoactive yellow protein photocycle investigated by time-resolved crystallography,” Biophys. J. 102, 325–332 (2012). 10.1016/j.bpj.2011.11.4021 22339869 PMC3260688

[73] Tsai, M. D. , Wu, W. J. , and Ho, M. C. , “ Enzymology and dynamics by cryogenic electron microscopy,” Annu. Rev. Biophys. 51, 19–38 (2022). 10.1146/annurev-biophys-100121-075228 34932913

[74] Weinberg, R. A. , *The Biology of Cancer* ( Garland Science, New York, 2007).

[75] Wilamowski, M. , Sherrell, D. A. , Kim, Y. , Lavens, A. , Henning, R. W. , Lazarski, K. , Shigemoto, A. , Endres, M. , Maltseva, N. I. , Babnigg, G. *et al.*, “ Time-resolved β-lactam cleavage by L1 metallo-β-lactamase,” Nat. Commun. 13, 7379 (2022). 10.1038/s41467-022-35029-3 36450742 PMC9712583

[76] Wilson, M. A. , “ Mapping enzyme landscapes by time-resolved crystallography with synchrotron and x-ray free electron laser light,” Annu. Rev. Biophys. 51, 79–98 (2022). 10.1146/annurev-biophys-100421-110959 34932909 PMC9132212

[77] Zimanyi, L. , Kulcsar, A. , Lanyi, J. K. , Sears, Jr., D. F. , and Saltiel, J. , “ Intermediate spectra and photocycle kinetics of the Asp96 → Asn mutant bacteriorhodopsin determined by singular value decomposition with self-modeling,” Proc. Natl. Acad. Sci. U. S. A. 96, 4414–4419 (1999a). 10.1073/pnas.96.8.4414 PMC16346 10200276

[78] Zimanyi, L. , Kulcsar, A. , Lanyi, J. K. , Sears, Jr., D. F. , and Saltiel, J. , “ Singular value decomposition with self-modeling applied to determine bacteriorhodopsin intermediate spectra: Analysis of simulated data,” Proc. Natl. Acad. Sci. U. S. A. 96, 4408–4413 (1999b). 10.1073/pnas.96.8.4408 PMC16345 10200275

